# Characterization of barley (*Hordeum vulgare *L.) NAC transcription factors suggests conserved functions compared to both monocots and dicots

**DOI:** 10.1186/1756-0500-4-302

**Published:** 2011-08-19

**Authors:** Michael W Christiansen, Preben B Holm, Per L Gregersen

**Affiliations:** 1 Department of Molecular Biology and Genetics, Aarhus University, Research Centre Flakkebjerg, 4200 Slagelse, Denmark

## Abstract

**Background:**

The NAC transcription factor family is involved in the regulation of traits in both monocots and dicots of high agronomic importance. Understanding the precise functions of the NAC genes can be of utmost importance for the improvement of cereal crop plants through plant breeding. For the cereal crop plant barley (*Hordeum vulgare *L.) only a few *NAC *genes have so far been investigated.

**Results:**

Through searches in publicly available barley sequence databases we have obtained a list of 48 barley *NAC *genes (*HvNACs*) with 43 of them representing full-length coding sequences. Phylogenetic comparisons to Brachypodium, rice, and Arabidopsis NAC proteins indicate that the barley NAC family includes members from all of the eight NAC subfamilies, although by comparison to these species a number of *HvNACs *still remains to be identified. Using qRT-PCR we investigated the expression profiles of 46 *HvNACs *across eight barley tissues (young flag leaf, senescing flag leaf, young ear, old ear, milk grain, late dough grain, roots, and developing stem) and two hormone treatments (abscisic acid and methyl jasmonate).

**Conclusions:**

Comparisons of expression profiles of selected barley *NAC *genes with the published functions of closely related *NAC *genes from other plant species, including both monocots and dicots, suggest conserved functions in the areas of secondary cell wall biosynthesis, leaf senescence, root development, seed development, and hormone regulated stress responses.

## Background

In recent years, research in the regulatory roles of members of the plant-specific NAC (NAM, ATAF-1,2, CUC) transcription factor family has increased considerably. It has become evident that these transcription factors are essential components in the regulation of a multitude of traits in plants, including traits of agronomic importance such as development, senescence, tolerance to both biotic and abiotic stresses, and hormone responses.

The first NAC transcription factor was described fifteen years ago by Souer *et al*. [1]. Since then the genomes of a number of plant species have been fully sequenced, revealing the *NAC *gene family to code for one of the largest families of transcription factors in plants [2]. Members of the family are identified by the presence of the NAC domain [3]. This domain consists of five subdomains: A-E [4,5] that make up motifs for both DNA-binding and protein-protein interactions [6].

**Figure 1:**
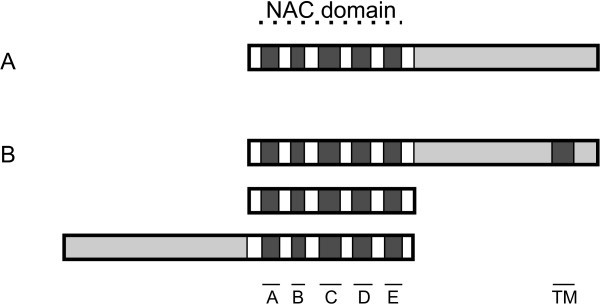
**Structure of the NAC transcription factor**. **A**: Typical NAC transcription factor, with N-terminal NAC domain, consisting of five conserved subdomains, A-E. The C-terminal part of the protein is more variable and contains the transcriptional activation or repressing region. **B**: Several variations on the typical NAC transcription factors can be found. A number of characterized NAC proteins have a conserved transmembrane motive, TM, in the far C-terminal region. Other variations include proteins with NAC domain only or proteins with the NAC domain in the C-terminal.

A typical NAC transcription factor has the conserved NAC domain in the N-terminal [5] as well as a more variable, transcriptional activation or repression region in the C-terminal [7] (figure 1A). Thirteen Arabidopsis and six rice NAC transcription factors have been shown to contain an α-helical transmembrane motif in the far C-terminal region which anchors the NAC protein to intracellular membranes rendering them inert. Only through controlled proteolytic cleavage from this anchor are the proteins able to exert their function [8]. Examination of known *NAC *families reveals a few atypical *NAC *genes, such as genes encoding only the NAC domain or genes with the NAC domain in the C-terminal with the variable region preceding it (figure 1B). None of these atypical *NAC *genes have been among closely characterized *NAC *genes, and therefore their functions are still unknown.

There have been several attempts to classify the phylogenetic relationships among all members of the NAC family [2,5,9,10]. However, the most comprehensive classification to date is the one published by Shen *et al*. [2], who divided the NAC superfamily into eight distinct NAC-subfamilies, NAC-a to NAC-h, based on NAC domain alignments. Furthermore, each subfamily was divided into several subgroups based on C-terminal motifs.

Since the first *NAC *gene was described, a number of NAC transcription factors have been characterized, in particular from the Arabidopsis NAC family (ANACs, 115 members) and, to a lesser extent, from the rice NAC family (ONACs, 144 members). There have been only a few detailed studies on *NAC *genes from species with non-sequenced genomes, such as wheat and barley [11,12].

So far it is known that many aspects of secondary cell wall biosynthesis (SCWB) are regulated by NAC transcription factors. Arabidopsis NST1 (ANAC043) was found to promote secondary cell wall thickening in the stem fibers [13-15]. NST2 (ANAC066), a close homologue of NST1, seems to have some functional redundancy to NST1. However, it shows strong promoter activity in the anthers, suggesting this tissue to be the primary site of NST2 regulation [15]. A homologue of NST1 in *Medicago truncatula *(MtNST1) was recently found to regulate lignin biosynthesis in the interfascicular region of the cell wall [16]. SND1 (NST3/ANAC012) appears to specifically regulate xylary fiber thickness [13,14,17,18]. This regulation most likely involves SND2 and SND3 (ANAC073 and ANAC010, respectively), which are both downstream targets of SND1 [19]. In Populus an orthologue of SND2 and SND3, PopNAC154, was shown to affect stem elongation [20].

Several NACs have been shown to regulate leaf senescence. Guo and Gan [21] described how AtNAP (ANAC029) could induce or delay leaf senescence in overexpression or knockout plants, respectively. More recently, the two paralogues ORE1 (AtNAC2/ANAC092) [22,23] and ORS1 (ANAC059) [24] were found to regulate salt-induced senescence. Positional cloning of a quantitative trait locus associated with increased grain protein content in wheat lead to the discovery of the wheat NAC transcription factor NAM-B1 [11]. As a possible orthologue of AtNAP NAM-B1 was also able to induce leaf senescence as well as increase grain protein content [25]. Besides its role in leaf senescence, ORE1 also plays a part in lateral root development as this formation is promoted in transgenic plants overexpressing ORE1 [26]. Another NAC transcription factor, NAC1 (ANAC021), has similarly been shown to induce the formation of lateral roots [27].

So far a number of NAC transcription factors have been shown to be involved in biotic and abiotic stress responses. In particular, tolerance to drought stress has been the center of many studies. Abscisic acid (ABA) accumulation seems to be a major trigger of plant genetic drought responses [28], which is also reflected by the fact that all the NAC transcription factors reported to confer drought tolerance are inducible by ABA treatment. In rice, this applies for SNAC1 (ONAC002) [29], ONAC045 [30], OsNAC6 (SNAC2/ONAC048) [31], OsNAC52 (ONAC088) [32], and OsNAC10 (ONAC120) [33], and in Arabidopsis it applies to ATAF-1 (ANAC002) [34], ANAC019 [35], ANAC055 [35], NTL6 (ANAC062) [8], and RD26 (ANAC072) [35,36].

Jasmonic acid (JA) is another phytohormone known to be involved in various stress responses, particularly in response to pathogen attacks [37]. This is also reflected in the number of reported NACs that have a role in pathogen defense and are induced by JA: GRAB2 [38], RIM1 (ONAC054) [39], ANAC019 [40], ANAC055 [40], and ATAF-2 (ANAC081) [41].

Untill now, only five barley *NAC *genes have been mentioned in the literature. The first study was done in 2003 by Scharrenberg *et al*. [42] who isolated cDNA clones from senescing barley flag leaves. One of these clones, named *HvSF6*, showed homology to wheat NAC transcription factor *GRAB2 *earlier reported to be involved in wheat Geminivirus defense [38]. The expression of this *NAC *gene was found to be induced by both age-dependent and dark-induced senescence, as well as a combination of the hormones ethylene and JA. Interestingly, neither hormone alone was enough to cause the induction of this gene.

Robertson [43] identified, through yeast one-hybrid screens, a barley NAC transcription factor, HSINAC, interacting with HvSPY. HvSPY is a negative regulator of gibberellin signaling. This *NAC *gene was found to be highly expressed in shoot, mature blade, sheath, and mature aleurone, and lowly expressed in young blade and stem, and was barely detectable in coleoptile and root. Jensen *et al*. [44] published their results on the barley HvNAC6 transcription factor, a homologue of Arabidopsis ATAF-1. They presented evidence of a role for HvNAC6 in penetration resistance to powdery mildew fungus. Besides being up-regulated during powdery mildew infection, no other expression data was presented.

Ogo *et al*. [45] published a comprehensive study on a rice NAC transcription factor called IDEF2. IDEF2 was suggested to be involved in iron homeostasis and was identified by its specific binding to promoter DNA containing an iron deficiency response element (IDE). A barley IDE was used in the same way to identify a barley homologue of IDEF2 called HvIDEF2. It was isolated from a root cDNA library, but otherwise no expression data was presented.

Finally, there have been two publications on the barley *HvNAM-1 *[12,46] encoding a homologue of the wheat TtNAM-B1 NAC transcription factor shown to regulate both senescence and grain protein content (GPC) [11]. Gene polymorphisms of *HvNAM-1 *in three Hordeum species explained some of the variation in GPC [12] suggesting that the biological function of HvNAM-1 is similar to that of TtNAM-B1.

Barley (*Hordeum vulgare *L.) is an important food and feed crop worldwide. Besides its agronomic importance, barley has also been used as the model species of choice for many researchers in the field of small grain cereal crops. Although its genome has not yet been fully sequenced, an extensive EST (expressed sequence tags) collection is available containing samples from a wide variety of tissues, different developmental stages, and pathogen infections, as well as an increasing amount of microarray data from the Affymetrix Barley1 GeneChip (http://www.plexdb.org/plex.php?database=Barley). Considering that many of the NAC transcription factors in barley will most likely turn out to be key regulators of important agronomic traits, as they are in other species, studies of this gene family are pertinent.

This work presents the first phylogenetic classification of *H. vulgare NAC *genes (*HvNACs*). 48 individual *HvNACs *were identified with 43 of them representing full-length coding sequences. Specific, quantitative real-time PCR (qRT-PCR) primers could be designed for 46 *HvNAC *genes, and the expression profiles of these were investigated in eight tissues of the barley plant as well as after two different hormone treatments of leaves with ABA and methyl jasmonate (MeJA). In conclusion, the results provide good evidence that also in barley the *NAC *gene family is involved in regulatory pathways of importance for agronomic traits.

## Results

### *HvNAC *genes

Searching all presently available barley nucleotide sequences for features of the characteristic NAC domains resulted in a list of 48 barley *NAC *genes either based on full-length cDNA, genomic sequences, or EST contigs. Nine of the genes were represented by only partial coding sequences based on homologous proteins in Brachypodium and rice. In order to obtain more full-length coding sequences (CDS), partial length EST clones were resequenced or cloned and sequenced when possible. This procedure reduced the number of partial length genes from 9 to 5. The complete list of established barley *NAC *genes is shown in table 1. The barley *NAC *genes were named according to the recommendations by Gray *et al*. [47]. The numbering of the genes was arbitrary except for a few sequences, e.g. *HvNAC001 *and *HvNAC006*, where annotation had already been made for submitted sequences. Sequence alignments of the HvNACs and all NAC proteins known to contain a C-terminal transmembrane anchor revealed four barley NACs with this motif: HvNAC002, HvNAC007, HvNAC016 and HvNAC048.

**Table 1:** Barley HvNACs

Gene	Accession#	Length	Group	Putative orthologues (percent identity)
HvNAC001	AK250475	Full	d-8	BdNAC067(91), ONAC060(84)
HvNAC002	AK249396	Full	b-2	BdNAC048(82), ONAC040(77)
HvNAC003	AK249102	Full	a-8	BdNAC001(85), ONAC002(81)
HvNAC004	AM500853	Full	a-8	BdNAC041(82), ONAC068/OsNAC4(75)
HvNAC005	AK251058	Full	a-6	BdNAC023(82), ONAC058(70)
HvNAC006/HvNAC6 [44]	AM500854	Full	a-9	BdNAC043(85), ONAC048/OsNAC6(83), ANAC002/ATAF1(65)
HvNAC007	AK249749	Full	b-6	BdNAC052(73), ONAC037(62)
HvNAC008/HvSF6 [66]	FR821737	Full	d-9	ONAC015(60)
HvNAC009	FR819761	Full	d	no close homologues
HvNAC010	FR821754	Full	f-2	BdNAC080(75), ONAC001(60)
HvNAC011	AK251493	Full	b-10	BdNAC081(93), ONAC109(90), ANAC057(72)
HvNAC012	FR819762	Full	e-4	BdNAC044(80), ONAC075(76), ANAC034/LOV1(51)
HvNAC013	AK376297	Full	d-9	BdNAC024(84), ONAC039(74)
HvNAC014	FR821738	Full	d-10	BdNAC093(77), ONAC004/OsNAC2(77)
HvNAC015	FR821739	Full	d-8	BdNAC053(78), ONAC104(76)
HvNAC016	AK366470	Full	b-2	BdNAC065(75), ONAC070(69)
HvNAC017	FR821740	Full	d	no close homologues
HvNAC018	FR821741	Full	d	no close homologues
HvNAC019	FR819764	Partial	d	-
HvNAC020	FR821742	Full	a-9	BdNAC071(88), ONAC009/OsNAC5(82), ANAC081/ATAF2(57)
HvNAC021	AK370287	Full	d-2	BdNAC003(90), ONAC006(73)
HvNAC022	AK365398	Full	d-8	BdNAC009(87), ONAC011(82), ANAC022(51)
HvNAC023	FR821745	Full	a-6	BdNAC005(77), ONAC103(73)
HvNAC024	FR821746	Full	d	no close homologues
HvNAC025	AK364002	Full	d-7	ONAC032 (63)
HvNAC026	FR819767	Full	a-4	BdNAC091(83), ONAC079(79), ANAC104/XND1(56)
HvNAC027	AK368213	Full	a-6	BdNAC090(70), ONAC131(63)
HvNAC028/IDEF2 [67]	AB362161	Full	b-3	BdNAC032(72), ONAC036(64)
HvNAC029/HvNAM-1 [12]	EU908210	Full	a-5	BdNAC006(81), ONAC010(72)
HvNAC030	DQ869679	Full	a-5	BdNAC006(81), ONAC010(73)
HvNAC031/HSINAC [43]	AY672069	Full	e-4	BdNAC050(79)
HvNAC032	AK248480	Full	d-7	BdNAC096(82), ONAC008(76)
HvNAC033	AK248449	Full	c-3	ONAC029(79), BdNAC051(73), ANAC043/NST1(57)
HvNAC034	AK249120	Full	c-3	ONAC007(72)
HvNAC035	FR821748	Full	b-4	BdNAC029(77), ONAC074/OsNAC8(61)
HvNAC036	AL505464	Partial	d-9	-
HvNAC037	AK371156	Full	d-9	no close homologues
HvNAC038	BY847894	Partial	c-4	-
HvNAC039	AK370035	Full	g-9	BdNAC038(91), ONAC073(84)
HvNAC040	AK361879	Full	h-3	no close homologues
HvNAC041	FR821751	Full	f-2	BdNAC061(68), ONAC005(62)
HvNAC042	AK361273	Full	h	BdNAC007(70)
HvNAC043	GH216054	Partial	h-5	-
HvNAC044	AK364683	Full	f-3	ONAC041 (65)
HvNAC045	BF259201	Full	h-5	no close homologues
HvNAC046	AK252960	Full	h	BdNAC076(90)
HvNAC047	CV057263	Partial	h	-
HvNAC048	AK355552	Full	b-1	ONAC042(63)

List of barley HvNACs with EMBL/GenBank accession numbers. The length of each established gene transcript is given as either full or partial coding sequence as compared to wheat or Brachypodium homologues. Phylogenetic grouping was performed according to Shen *et al*. [2] and was based only on the NAC domain of the encoded protein. For HvNACs with no close rice or Arabidopsis homologues, only the subfamily is noted. The putative orthologues are based on full-length protein alignments and the presence of conserved motifs in the C-terminal. No orthologues are listed for the partial length proteins. The percent identity, on full-length amino acid level, between the barley NAC protein and the orthologue in question is given in parenthesis. Numbers in square brackets in the gene name column represent references in the bibliographic reference list.

### Phylogenetics

**Figure 2:**
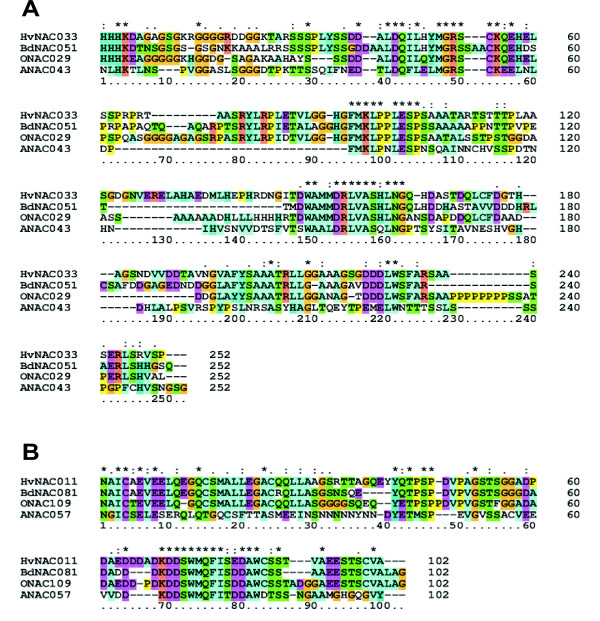
**Conserved C-terminal domains**. A: C-terminal amino acid (aa) alignment of HvNAC033 and its closest homologues from Brachypodium, rice, and Arabidopsis. Three different characters are used to indicate conservation: '*' indicating full conservation of aa, ':' indicating strong conservation (non-identical aa, but with highly similar physico-chemical properties) and '.' indicating weak conservation (non-identical aa, but with somewhat similar physico-chemical properties). **B**: Same as A, but for HvNAC011 and its homologues.

The HvNACs were classified according to the system proposed by Shen *et al*. [2] based on positions in a phylogenetic tree made from the alignment of the conserved NAC domains of ONACs, ANACs, and HvNACs (additional file 1). The phylogenetic tree also includes *Brachypodium distachyon *NACs (BdNACs) as well a few selected NACs from other species discussed in this paper. Table 2 shows the distribution of HvNACs among the eight NAC subfamilies. In addition to the NAC subfamily classification, Shen *et al*. [2] further divided the NAC transcription factors into subgroups based on homology of C-terminal regions. By referring to the subgrouping of the ANACs and ONACs, each of the full-length HvNACs could in most cases be assigned to a NAC subgroup as well. These subgroups are listed in table 1. This table also lists putative orthologues of each HvNAC among BdNACs, ONACs, and ANACs. Through full-length protein alignments, the possible orthologues were identified as having a highly conserved NAC domain as well as one or more conserved C-terminal motifs. Figure 2 illustrates the identification of C-terminal motifs for putative orthologues exemplified by HvNAC033 and HvNAC011. In both cases at least three conserved, smaller motifs can be observed. After identifying each of the possible orthologues through visual inspection of full-length protein alignments, their percent identity was calculated as noted in table 1.

**Table 2:** NAC gene distribution

Subfamily	HvNAC	BdNAC	ONAC	SbNAC	ANAC
A	10	18	23	20	17
B	7	11	12	12	35
C	3	9	10	10	13
D	16	12	23	21	17
E	2	13	15	13	9
F	3	7	10	3	5
G	1	8	15	15	15
H	6	18	36	19	4

Total	48	96	144	113	115

Distribution of NAC genes from barley, Brachypodium, rice, sorghum, and Arabidopsis in the groups proposed by Shen *et al*. [2].

### *HvNAC *gene expression studies

**Figure 3:**
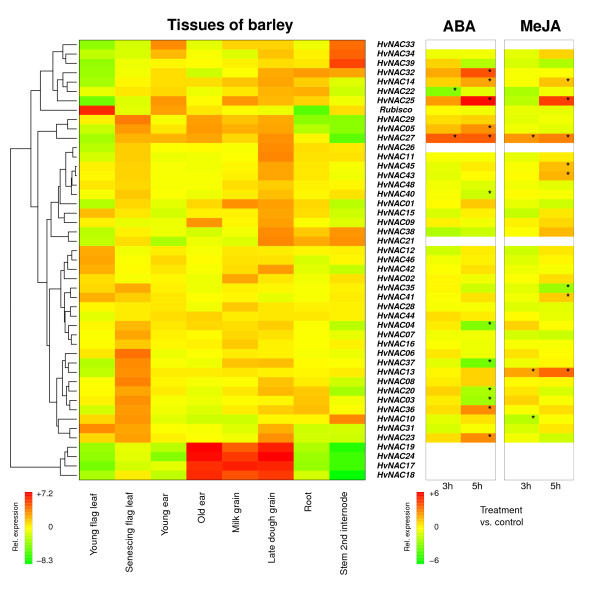
**HvNAC gene expression patterns**. Heatmaps showing the gene expression patterns across different tissues of the barley plant and following 3 or 5 hours of treatment with the plant hormones abscisic acid (ABA) or methyl jasmonate (MeJA) for 46 *HvNAC *genes and *Rubisco *(small subunit) as control gene. Relative gene expression levels at the log2 scale are indicated by a color scale, where red indicates high and green low expression. The color bars in the lower corners show the range of gene expression differences in the two types of experiments. White color in the hormone experiment indicates excluded *HvNAC *genes with expression data of poor quality (aberrant dissociation curves at low expression levels). The gene expression levels were determined by quantitative real-time PCR as described in the material and methods section. In brief, the presented expression data are based on three (tissues) or two (hormone treatments) biological replicates, each with three technical replicate PCR runs, followed by normalization to the expression of the *18 S ribosomal RNA *gene. Prior to construction of the heatmap for the tissue experiment, the relative expression values for each gene were re-scaled to have mean equal to zero. Asterisks in the hormone experiments indicate significant differences at the 0.05 level between treatment and control (p-values adjusted for multiple comparisons according to default settings in the Limma software).

In order to extend our characterization of the HvNACs, tissue-specific gene expression studies using qRT-PCR were performed. One of the partial length sequences *HvNAC047 *was excluded from this experiment, since the design of a specific set of qRT-PCR primers was not possible. *HvNAC030 *had aberrant expression, based on the dissociation curves, in most of the tissues investigated, and so it was excluded after analysis of the qRT-PCR data. The individual tissues were selected to cover a range of plant organs. As several NAC genes from rice and Arabidopsis have been shown to be hormone and stress responsive, treatments with the two stress hormones ABA and MeJA were included in the expression studies as well. The data collected from these experiments are presented in figure 3. The raw data from the qRT-PCR experiment, as well as calculated p-values, can be found in additional file 2. The relative expression levels in each tissue were determined using the Limma software with non-senescing flag leaf tissue as the reference. Prior to construction of the heatmap for the tissue experiment in figure 3, the relative expression values for each gene were re-scaled to have mean equal to zero in order to bring the different expression patterns into the same colour range centred on zero. We have included in the figure one non-*NAC *gene *Rubisco*, encoding the Rubisco small subunit, as a control that reflects the amount of green photosynthesizing tissue. From figure 3* Rubisco *is, as expected, found to be up-regulated in young flag leaf, young ear and stem, and strongly down-regulated in roots.

The eight tissues were selected in order to identify *HvNAC *genes associated with processes already known to be NAC regulated in other species. SCWB is represented here by the elongating stem tissue, and we see a small group of just three genes (*HvNAC033, HvNAC034*, and *HvNAC039*) clearly up-regulated. Leaf senescence is represented by the senescing flag leaf, where a number of genes are up-regulated. If we observe the old ear and late dough grain as tissues, where the senescence process is also taking place, as well as ABA induction, since ABA is a known senescence inducer [48], three genes stand out as putative senescence regulators: *HvNAC005, HvNAC027*, and *HvNAC029*. A few NAC transcription factors regulate root development, a process represented here by the root tissue. Only three genes are highly up-regulated in this tissue: *HvNAC021, HvNAC022*, and *HvNAC032*. The old ear, milk grain, and late dough grain all represent various stages of seed development and as such could help identify genes involved with regulation of this. Looking at these tissues together, four genes stand out quite clearly: *HvNAC017, HvNAC018, HvNAC019*, and *HvNAC024*.

In the hormone treatment experiment, seven genes were excluded from the heatmap due to expression data of poor quality (aberrant dissociation curves at low expression levels). The non-senescing flag leaf samples showed low expression for all of the excluded genes, and the poor quality might have been caused by an even lower expression in the used plant material, which was from young flag leaves harvested at an earlier developmental stage than the non-senescing flag leaves in order to avoid environmental stresses. With the two hormone treatments it is noticeable that only a few genes were up- or down-regulated after three hours of treatment, whereas more significant changes were observed after five hours. There appears to be some phylogeny correlations in the genes induced by ABA. All members of the NAC-a-6 subgroup, *HvNAC005, HvNAC023*, and *HvNAC027*, are significantly up-regulated by ABA, whereas both members of the closely related NAC-a-8 subgroup, *HvNAC003 *and *HvNAC004*, are down-regulated. All members of the NAC-d-7 subgroup, *HvNAC025 *and *HvNAC032*, are up-regulated as well. The remaining ABA-induced genes do not seem to be systematically induced, in terms of phylogeny, which seems also to be the case for all the MeJA-induced genes. Of all the genes *HvNAC027 *stands out as being highly responsive to both hormones.

Data from experiments using the Affymetrix 22 K Barley1 GeneChip provide a wealth of information on gene expression in barley (http://www.plexdb.org/plex.php?database=Barley). However, comparisons of our data to Barley1 GeneChip data are complex, since, first, two important tissues in our study the naturally senescing flag leaf and the developing stem are not included in the Barley1 GeneChip experiment on development stages in barley [49], and, second, only 20 of the *HvNAC *genes are represented by near full-length contigs of the Barley1 GeneChip. The remaining *NAC *genes are represented by contigs of variable lengths or are missing (19). Hence, we decided to omit here systematic comparisons of our data to Barley1 GeneChip data. However, several *HvNAC *genes did in fact show good correspondence among our results and Barley1 GeneChip data. Examples from experiments BB3 and BB80 are: Up-regulation of *HvNAC017, HvNAC18*, and *HvNAC024 *in the developing seed (Contig8993_at, Contig9284_at, and Contig11340_at), up-regulation of *HvNAC021 *and *HvNAC022 *in the root (Contig15867_at and Contig6484_at), up-regulation of *HvNAC033 *in young ear/floral bracts (Contig19673_at), and up-regulation of *HvNAC005 *and *HvNAC027 *by ABA treatment (Contig14026_at and HM07L17r_at).

## Discussion

### HvNACs

In this study we have identified 48 HvNACs. However, by observing the distribution of HvNACs across the eight NAC subfamilies in table 2, it is evident that these 48 genes do not represent the entire barley NAC family. Most likely, we have only identified about half of the members of the complete barley NAC family. Taking into account the distribution in table 2, the majority of the missing barley NAC genes will most likely come from subfamily H as well as E and G. As the available barley EST databases are already quite extensive, it seems unlikely that many of these missing *HvNAC *genes will be identified before the barley genome is fully sequenced and made available. Again, as the barley EST databases are extensive, it could be argued that the most essential HvNACs with regard to plant development, abiotic and biotic stress responses are probably among the 48 genes identified in this study. However, a gene with rare or very low expression does not equal a non-essential product, and thus important NAC genes could still be absent from EST collections.

### Phylogenetics

A number of different suggestions for the classification of NAC transcription factors have been reported [2,5,9,10]. The most comprehensive one by Shen *et al*. [2] was adopted in this study on barley *NAC *genes.

For identification of putative Brachypodium, rice, and Arabidopsis orthologues, full-length protein sequences with closely related NAC domain regions were aligned to reveal the C-terminal motifs. Generally, there is low conservation in the C-terminal region of the NAC proteins, however, when aligning orthologues across species, the presence of small C-terminal motifs becomes apparent. Table 1 shows putative orthologues, as well as their percent identity of each HvNAC, based on both a highly conserved N-terminal NAC domain and on conserved motifs in the C-terminal. Seven *HvNAC *genes encode both a NAC domain and C-terminal motifs conserved throughout Brachypodium, rice, and Arabidopsis. The orthologous NAC proteins among the grasses are highly similar, often sharing with barley more than 70% (for Brachypodium) or 60% (for rice) of their amino acid sequence. It was not possible to identify clear Arabidopsis orthologues for many of the HvNACs. However, there were a few which showed high conservation. In these cases the percent identity was above 50%. It could be speculated that NAC genes showing conservation throughout the monocots and dicots could be involved in important regulatory pathways. In support of this, five out of the seven Arabidopsis NAC proteins identified as HvNAC orthologues have been characterized in the literature and in all cases found to have profound effects on the plants if overexpressed or knocked out [13,41,50-52].

### *HvNAC *expression

To validate our expression data, we included *Rubisco *as an internal control, which showed an expression pattern as expected, however, further validation can be done by comparing our results with previous reports. *HvSF6 *(*HvNAC008*) was found to be up-regulated in senescing tissue [42], matching entirely with our data. *HvSF6 *was further seen to be induced by a combination of ethylene and MeJA, but not MeJA alone [42], which also agrees with the lack of induction by our MeJA treatment. *HSINAC *(*HvNAC031*) was found to be up-regulated in mature blade and mature aleurone [43], fitting well with the up-regulation we observed in the senescing leaf and late dough grain, respectively. Furthermore, a low expression in young blade and stem was reported. We also observed low expression in the stem tissue, however, our young flag leaf tissue showed relatively high expression. It is possible that our young, but fully developed, flag leaf sample resembles their mature blade more than their young blade. Finally, Robertson [43] reported barely detectable expression levels in the roots, while we observed root levels similar to the low expression in the stem.

No expression patterns were reported for *HvIDEF2 *(*HvNAC028*), besides it being isolated from a root cDNA library [45]. Interestingly, this gene was one of the most stably expressed genes across all eight tissues. Likewise, no *HvNAM-1 *(*HvNAC029*) induction was reported [12,46]. However, we did observe a significant induction in the senescing flag leaf, which would be expected for an orthologue of TtNAM-B1.

The overall results presented in figure 3 emphasize the suggested involvement of NAC transcription factors as regulatory factors in a range of processes during plant development and stress responses. We will focus here on a few selected genes and discuss their expression patterns in comparison to the known and suggested functions of phylogenetically related *NAC *genes.

SCWB has been the focus of several *NAC *gene studies, and many *NAC *genes have been shown to regulate aspects of this process. *HvNAC039 *is clearly up-regulated in the developing stem tissue, where extensive SCWB is expected to take place. Considering that HvNAC039 belongs to subgroup NAC-g-9, as do both SND2, SND3 (ANAC073 and ANAC010, respectively), and PopNAC154, a role in SCWB is not unlikely for this NAC gene. *HvNAC033 *and *HvNAC034 *are also up-regulated in the stem tissue and may thus regulate aspects of the SCWB as well. Protein alignments identified HvNAC033 as a possible orthologue of both NST1 (ANAC043) and MtNST1. Furthermore, considering the expression data, HvNAC033 is very likely an NST orthologue. HvNAC034 is very closely related to HvNAC033, both belonging to subgroup NAC-c-3, and therefore also related to NST1 and MtNST1. Both *HvNAC033 *and *HvNAC034 *are also up-regulated in the developing young ear, where a high degree of SCWB is also expected to take place, supporting the hypothesis that they could be regulators of this process.

Leaf senescence has also been the focus of several *NAC *gene studies. There are several *HvNACs *which are up-regulated in the senescing flag leaf, however, by including the relative expression levels across the old ear and late dough grain tissues, three genes stand out: *HvNAC005, HvNAC027*, and *HvNAC029*. HvNAC029 is an orthologue of NAM-B1 from wheat and has furthermore been mapped to a quantitative trait locus (QTL) for leaf senescence in barley [46], clearly supporting the hypothesis of a role for this transcription factor in leaf senescence regulation. HvNAC005 and HvNAC027 both belong to subgroup NAC-a-6, as does the Arabidopsis senescence regulator AtNAP (ANAC029), suggesting a possible link to senescence. Both are also found to be up-regulated following ABA treatment, adding further evidence, as ABA is known to be an inducer of senescence [48].

Three highly up-regulated *HvNAC *genes were found in the root tissue: *HvNAC021, HvNAC022*, and *HvNAC032*. This could indicate roles in root development, especially as they all belong to subfamily NAC-d, as do both NAC1 (ANAC021) and ORE1 (ANAC092). Although it is possible that all three are involved in regulating root development, HvNAC022 stands out as being phylogenetically very closely related to NAC1 as well as being down-regulated in the presence of ABA, which is known to have an inhibitory effect on lateral root formation [53]. Using the online GeneVestigator software (http://www.genevestigator.com/gv/), it was confirmed that Arabidopsis NAC1 is also down-regulated by ABA in an experiment very similar to the one presented here (Experiment ID: AT-00231). These results support the hypothesis that at least HvNAC022 is a putative regulator of root development.

HvNAC017, HvNAC018, HvNAC019 and HvNAC024 form, together with HvNAC009, a small barley-specific group of NAC transcription factors with no clear orthologues in other plant species investigated so far. The gene expression patterns in the data presented here indicate that these four genes could regulate aspects of seed development. This hypothesis might be supported by results of Guo *et al*. [54] and Verza *et al*. [55]. They reported on the expression of two maize NAC genes *ZmNRP-1 *and *ZmAPN-1*, respectively. Both of these genes are closely related to the barley specific group, although they do not appear as direct orthologues. It was found that *ZmNRP-1 *had endosperm specific expression [54] and that *ZmAPN-1 *expression was restricted to the aleurone cell layer [55].

Many *NAC *genes that have been found to be involved in various forms of stress responses and tolerances, and very often the same genes are also found to be induced by stress hormones. In order to get an initial overview of the involvement of the HvNAC family in stress regulation, we tested the effects of treatment with the two stress hormones ABA and MeJA on *HvNAC *expression. Notably, most of the significant changes happened after five hours of treatment. This could either be due to a secondary wave of gene induction or to generally slow inductions, perhaps related to our experimental setup. Another observation from our data is that some of the ABA-induced genes were phylogenetically related, i.e. belong to the same subgroup. *HvNAC027 *stood out as being highly responsive to both hormones, suggesting it could have a putative role in stress tolerance regulation. *TaNAC69 *from wheat, a direct orthologue of *HvNAC027*, displays a similar induction from ABA as well as induction by drought and cold [56].

## Conclusions

In this work we have compiled available sequences from public databases that represent 48 members of the *NAC *gene family in barley. The list of barley *NAC *genes is, evidently, not exhaustive, since a number of additional members exist in *NAC *gene families of the closely related species Brachypodium and rice.

From the data presented here, we have identified possible regulators of secondary cell wall synthesis (HvNAC033, HvNAC034, and HvNAC039), leaf senescence (HvNAC005, HvNAC027, and HvNAC029), and root development (HvNAC022). Furthermore, we have identified putative regulators of processes in seed development (HvNAC017, HvNAC018, HvNAC019).

The results presented here support the idea that the functional roles of NAC transcription factors are conserved throughout both monocot and dicot species. Furthermore, they clearly demonstrate that the qRT-PCR approach for an initial characterization of the barley NAC family was a good choice. Most of the genes selected as putative regulators of particular processes, based on the qRT-PCR results, were either direct orthologues of, or at least very closely related to, *NAC *genes from other species, specifically known to regulate these processes.

## Methods

### Identification of HvNACs

All publicly available barley nucleotide sequences were collected from the Nucleotide, GSS, EST and UniGene databases of the National Center for Biotechnology Information (NCBI) (http://www.ncbi.nlm.nih.gov/) and Plant Genomic Database (http://www.plantgdb.org/). These sequences were searched for the presence of barley *NAC *gene sequences using the tBLASTn algorithm. The NAC domain of all available *Brachypodium distachyon *and *Oryza sativa *NAC protein sequences were used as input for the BLAST function. Contig assembly was performed using the CAP3 algorithm [57]. As the vast majority of the collected sequences were in the form of expressed sequence tags (ESTs), each contig was manually inspected due to the error prone nature of ESTs. In the case that a contig was considered to be erroneous based on the translated protein sequence, all ESTs of the contig were carefully inspected and the contig manually corrected, if applicable. In order to obtain more full-length CDS sequences, partial length EST clones were re-sequenced or cloned and sequenced when possible. Primers used for cloning were based on homologous wheat or Brachypodium sequences.

### Phylogenetic analysis

All phylogenetic analyses were performed on the NAC domain part of each gene. Each HvNAC protein sequence was manually truncated immediately following the E subdomain as defined in Ooka *et al*. [5]. Sequences lacking a defined E subdomain were truncated based on multiple alignments of all HvNACs proteins. All alignments and phylogenetic trees were made using ClustalX 2.1 [58]. The phylogenetic tree in additional file 1 was drawn using the ape add-on package [59] of the R software [60].

### Plant material and treatments

Barley plants, cv. Golden Promise, were grown in greenhouse soil plots. Artificial illumination was used for supplementation and for ensuring a day/night cycle of 16/8 hr. All sampling were done between 12 noon and 2 pm, and samples were frozen in liquid nitrogen immediately after being harvested. For all tissue samples, three biological replicates were harvested and processed in parallel. The samples were stored at -80°C until RNA extraction was performed. Plants from which the roots were sampled were grown in individual pots containing perlite (Nordisk Perlite, Denmark), and sampling was performed when the plantlets were at the three leaf stage.

Young flag leaf material was harvested at the heading stage when the leaf was fully developed, and senescing flag leaves when green leaf area was reduced to approximately 50%. Young ear material was harvested immediately after heading, and the old ear material when the seeds were close to full maturity. The milk and late dough grain material comprised individual grains in the early milk stage and late dough stage, respectively. The stem samples comprised the lower approximately 5-6 cm of culms from the second internode from the top harvested just prior to heading. Young, light green leaves from small plantlets were sampled for genomic DNA (gDNA) isolation used for qRT-PCR primer testing.

For treatments with ABA and MeJA, young flag leaves were taken just prior to heading. In order to encounter developmental differences, the basal 10 cm of the leaves was divided into six pieces that were distributed across different time points of the time-course study. Hormone treatments were done in 10 ml water containing 50 μM hormone (dissolved in DMSO) and 0.005% Triton-X 100. Controls were treated with 0.005% Triton-X 100 and 0.1% DMSO. Two replicate samples comprising five leaf pieces were taken for RNA isolation at 0, 1, 3, and 5 hours after the unset of hormone treatment. At 0 hours only the control sample was taken.

### RNA isolation and cDNA synthesis

RNA isolation was performed on approximately 100 mg of frozen homogenized material using the Spectrum™ Plant Total RNA kit (Sigma) according to the manufacturer's recommendations. First-strand cDNA synthesis was performed on 3-5 μg of RNA and a nonamer, random oligonucleotide primer (2.5 μM) by incubation at 65°C for 5 min followed by 10 min at RT in a volume of 18,4 μL. 200 U SuperscriptII (Invitrogen), 40 U RNAsin (Promega), 1 × FS Buffer (Invitrogen), 10 μM dichlorodiphenyltrichloroethane 1,4-dithiothreitol (DDT), and 2 mM dNTPs (GE Healthcare) were added to make a final volume of 30 μl, which was incubated for 1 hour at 42°C and 10 min at 70°C, followed by the addition of 70 μl of water.

### Genomic DNA isolation

DNA was extracted from 1-2 g of leaf material using a DNA extraction buffer consisting of 1% Sodium lauroyl sarcosinate (sarkosyl), 100 mM Tris-HCl pH 8.5, 100 mM NaCl, 10 mM ethylenediaminetetraacetic acid (EDTA), 2% Polyvinylpolypyrrolidone (PVPP) followed by standard phenol/chloroform/isoamyl-alcohol separation and ethanol precipitation. The DNA was dissolved in 500 μl R40 (40 ug/ml RNAse A in 1 × TE) on an orbital shaker at 4°C overnight.

### Primer design and efficiency

To ensure specificity of each *HvNAC *primer pair, the online Primique software [61] was used to design primer sets for qRT-PCR. All other primers were designed with Oligo Explorer 1.2 from Gene Link, Inc. qRT-PCR primer sequences are listed in additional file 3. All primers were tested for their efficiency on gDNA. gDNA was used instead of cDNA, since many of the *HvNAC *genes were expected to have low expression levels. As such, measures were taken to avoid intron spanning amplicons during the design of the primers. Four dilutions of gDNA were used in the testing: 1, 1/8, 1/64, and 1/512 as well as a zero template control. Average values for three technical replicates were plotted against the concentration, and the efficiency was calculated from the slope of the best linear regression, using the formula: efficiency = 10^(-1/slope) [62]. Efficiencies of 2 ± 0.2 were acceptable. Primers were also tested without any template to ensure that no primer dimer products would occur.

### qRT-PCR

Quantitative real-time PCR was performed in 384 well plates using the ABI Prism7900HT Sequence Detection System with the Power SYBR^® ^Green PCR master mix (Applied Biosystems) according to the manufacturer's recommendations. 0.5 μl of cDNA was used for each qRT-PCR reaction and all tests were performed with three technical replicates. For each qRT-PCR run a dissociation stage was included, in order to be used in quality assessments of each sample during data analysis. The threshold cycle (Ct) for each run was determined using the automatic settings for baseline and threshold level of the ABI Prism 7900HT SDS software.

### Analysis of qRT-PCR data

Ct values exported from the ABI Prism 7900HT SDS software were used as raw data for the analysis of qRT-PCR data. The R software [60] and the add-on packages HTqPCR [63] and Limma [64] were used for the manipulation and analysis of the Ct values, and for generation of heatmaps of gene expression data. qRT-PCR runs showing high variation among technical replicates were manually inspected, and clear outliers and runs with aberrant dissociation curves were excluded from the analysis. Several possible reference genes were tested for their stability across different tissues, using the tools in the R package SLqPCR [65]. Based on this analysis, the barley *18 S ribosomal RNA *gene was selected as the most stable reference gene to be used in the normalization of gene expression of *NAC *genes in different tissues and treatments.

## Competing interests

The authors declare that they have no competing interests.

## Authors' contributions

MWC carried out BLAST searches, phylogenetics, molecular cloning, design of primers, tissue expression studies, and drafted the manuscript. PLG designed the study and carried out the phylogenetics, hormonal expression studies, data presentations in R, as well as revisions to the manuscript. PBH conceived the study, and participated in its design and coordination as well as revisions to the manuscript. All authors have read and approved the final manuscript.

## Supplementary Material

Additional file 1 **Phylogenetic tree**. Suppl_file1_Phylogenetic_tree.pdf. Phylogenetic tree of all HvNACs, BdNACs, ONACs, ANACs, and a few selected NAC genes from other species discussed in the study. Click here for file

Additional file 2 **Data and statistics for qRT-PCR results**. Suppl_file2_qRT-PCR data. Normalized qRT-PCR data used for heatmap construction, including statistics on significance. Click here for file

Additional file 3 **qRT-PCR primers**. Suppl_file3_qRT-PCR primers. Primer sequences, presented in a 5' to 3' direction, for all qRT-PCR primers used in this study. Click here for file

## References

[B1] Souer E vanHouwelingen A Kloos D Mol J Koes R The no apical meristem gene of petunia is required for pattern formation in embryos and flowers and is expressed at meristem and primordia boundaries Cell 1996 85 159 170 10.1016/S0092-8674(00)81093-4 8612269

[B2] Shen H Yin YB Chen F Xu Y Dixon RA A Bioinformatic Analysis of NAC Genes for Plant Cell Wall Development in Relation to Lignocellulosic Bioenergy Production Bioenergy Research 2009 2 217 232 10.1007/s12155-009-9047-9

[B3] Aida M Ishida T Fukaki H Fujisawa H Tasaka M Genes involved in organ separation in Arabidopsis: An analysis of the cup-shaped cotyledon mutant Plant Cell 1997 9 841 857 10.1105/tpc.9.6.841 9212461 PMC156962

[B4] Kikuchi K Ueguchi-Tanaka M Yoshida KT Nagato Y Matsusoka M Hirano HY Molecular analysis of the NAC gene family in rice Molecular and General Genetics 2000 262 1047 1051 10.1007/PL00008647 10660065

[B5] Ooka H Satoh K Doi K Nagata T Otomo Y Murakami K Matsubara K Osato N Kawai J Carninci P Hayashizaki Y Suzuki K Kojima K Takahara Y Yamamoto K Kikuchi S Comprehensive analysis of NAC family genes in Oryza sativa and Arabidopsis thaliana DNA Research 2003 10 239 247 10.1093/dnares/10.6.239 15029955

[B6] Ernst HA Olsen AN Skriver K Larsen S Lo Leggio L Structure of the conserved domain of ANAC, a member of the NAC family of transcription factors Embo Reports 2004 5 297 303 10.1038/sj.embor.7400093 15083810 PMC1299004

[B7] Hao YJ Song QX Chen HW Zou HF Wei W Kang XS Ma BA Zhang WK Zhang JS Chen SY Plant NAC-type transcription factor proteins contain a NARD domain for repression of transcriptional activation Planta 2010 232 1033 1043 10.1007/s00425-010-1238-2 20683728

[B8] Kim SY Kim SG Kim YS Seo PJ Bae M Yoon HK Park CM Exploring membrane-associated NAC transcription factors in Arabidopsis: implications for membrane biology in genome regulation Nucleic Acids Research 2007 35 203 213 17158162 10.1093/nar/gkl1068 PMC1802569

[B9] Fang Y You J Xie K Xie W Xiong L Systematic sequence analysis and identification of tissue-specific or stress-responsive genes of NAC transcription factor family in rice Molecular Genetics and Genomics 2008 280 547 563 10.1007/s00438-008-0386-6 18813954

[B10] Nuruzzaman M Manimekalai R Sharoni AM Satoh K Kondoh H Ooka H Kikuchi S Genome-wide analysis of NAC transcription factor family in rice Gene 2010 465 30 44 10.1016/j.gene.2010.06.008 20600702

[B11] Uauy C Distelfeld A Fahima T Blechl A Dubcovsky J A NAC gene regulating senescence improves grain protein, zinc, and iron content in wheat Science 2006 314 1298 1301 10.1126/science.1133649 17124321 PMC4737439

[B12] Jamar C Loffet F Frettinger P Ramsay L Fauconnier ML du Jardin P NAM-1gene polymorphism and grain protein content in Hordeum Journal of Plant Physiology 2010 167 497 501 10.1016/j.jplph.2009.10.014 20005003

[B13] Zhong RQ Richardson EA Ye ZH Two NAC domain transcription factors, SND1 and NST1, function redundantly in regulation of secondary wall synthesis in fibers of Arabidopsis Planta 2007 225 1603 1611 10.1007/s00425-007-0498-y 17333250

[B14] Mitsuda N Iwase A Yamamoto H Yoshida M Seki M Shinozaki K Ohme-Takagi M NAC transcription factors, NST1 and NST3, are key regulators of the formation of secondary walls in woody tissues of Arabidopsis Plant Cell 2007 19 270 280 10.1105/tpc.106.047043 17237351 PMC1820955

[B15] Mitsuda N Seki M Shinozaki K Ohme-Takagi M The NAC transcription factors NST1 and NST2 of Arabidopsis regulate secondary wall thickenings and are required for anther dehiscence Plant Cell 2005 17 2993 3006 10.1105/tpc.105.036004 16214898 PMC1276025

[B16] Zhao QA Gallego-Giraldo L Wang HZ Zeng YN Ding SY Chen F Dixon RA An NAC transcription factor orchestrates multiple features of cell wall development in Medicago truncatula Plant Journal 2010 63 100 114 20408998 10.1111/j.1365-313X.2010.04223.x

[B17] Zhong RQ Demura T Ye ZH SND1, a NAC domain transcription factor, is a key regulator of secondary wall synthesis in fibers of Arabidopsis Plant Cell 2006 18 3158 3170 10.1105/tpc.106.047399 17114348 PMC1693950

[B18] Ko JH Yang SH Park AH Lerouxel O Han KH ANAC012, a member of the plant-specific NAC transcription factor family, negatively regulates xylary fiber development in Arabidopsis thaliana Plant Journal 2007 50 1035 1048 10.1111/j.1365-313X.2007.03109.x 17565617

[B19] Zhong R Lee C Zhou J McCarthy R Ye Z A battery of transcription factors involved in the regulation of secondary cell wall biosynthesis in Arabidopsis Plant Cell 2008 20 2763 2782 10.1105/tpc.108.061325 18952777 PMC2590737

[B20] Grant EH Fujino T Beers EP Brunner AM Characterization of NAC domain transcription factors implicated in control of vascular cell differentiation in Arabidopsis and Populus Planta 2010 232 337 352 10.1007/s00425-010-1181-2 20458494

[B21] Guo YF Gan SS AtNAP, a NAC family transcription factor, has an important role in leaf senescence Plant Journal 2006 46 601 612 10.1111/j.1365-313X.2006.02723.x 16640597

[B22] Kim JH Woo HR Kim J Lim PO Lee IC Choi SH Hwang D Nam HG Trifurcate Feed-Forward Regulation of Age-Dependent Cell Death Involving miR164 in Arabidopsis Science 2009 323 1053 1057 10.1126/science.1166386 19229035

[B23] Balazadeh S Siddiqui H Allu AD Matallana-Ramirez LP Caldana C Mehrnia M Zanor MI Kohler B Mueller-Roeber B A gene regulatory network controlled by the NAC transcription factor ANAC092/AtNAC2/ORE1 during salt-promoted senescence Plant Journal 2010 62 250 264 10.1111/j.1365-313X.2010.04151.x 20113437

[B24] Balazadeh S Kwasniewski M Caldana C Mehrnia M Zanor MI Xue GP Mueller-Roeber B ORS1, an H2O2-Responsive NAC Transcription Factor, Controls Senescence in Arabidopsis thaliana Molecular Plant 2011 4 346 360 10.1093/mp/ssq080 21303842 PMC3063519

[B25] Uauy C Brevis JC Dubcovsky J The high grain protein content gene Gpc-B1 accelerates senescence and has pleiotropic effects on protein content in wheat Journal of Experimental Botany 2006 57 2785 2794 10.1093/jxb/erl047 16831844

[B26] He XJ Mu RL Cao WH Zhang ZG Zhang JS Chen SY AtNAC2, a transcription factor downstream of ethylene and auxin signaling pathways, is involved in salt stress response and lateral root development Plant Journal 2005 44 903 916 10.1111/j.1365-313X.2005.02575.x 16359384

[B27] Xie Q Frugis G Colgan D Chua NH Arabidopsis NAC1 transduces auxin signal downstream of TIR1 to promote lateral root development Genes & Development 2000 14 3024 3036 10.1101/gad.852200 11114891 PMC317103

[B28] Shinozaki K Yamaguchi-Shinozaki K Molecular responses to dehydration and low temperature: differences and cross-talk between two stress signaling pathways Current Opinion in Plant Biology 2000 3 217 223 10837265

[B29] Hu HH Dai MQ Yao JL Xiao BZ Li XH Zhang QF Xiong LZ Overexpressing a NAM, ATAF, and CUC (NAC) transcription factor enhances drought resistance and salt tolerance in rice Proceedings of the National Academy of Sciences of the United States of America 2006 103 12987 12992 10.1073/pnas.0604882103 16924117 PMC1559740

[B30] Zheng X Chen B Lu G Han B Overexpression of a NAC transcription factor enhances rice drought and salt tolerance Biochemical and Biophysical Research Communications 2009 379 985 989 10.1016/j.bbrc.2008.12.163 19135985

[B31] Nakashima K Tran LSP Van Nguyen D Fujita M Maruyama K Todaka D Ito Y Hayashi N Shinozaki K Yamaguchi-Shinozaki K Functional analysis of a NAC-type transcription factor OsNAC6 involved in abiotic and biotic stress-responsive gene expression in rice Plant Journal 2007 51 617 630 10.1111/j.1365-313X.2007.03168.x 17587305

[B32] Gao F Xiong AS Peng RH Jin XF Xu J Zhu B Chen JM Yao QH OsNAC52, a rice NAC transcription factor, potentially responds to ABA and confers drought tolerance in transgenic plants Plant Cell Tissue and Organ Culture 2010 100 255 262 10.1007/s11240-009-9640-9

[B33] Jeong JS Kim YS Baek KH Jung H Ha SH Do Choi Y Kim M Reuzeau C Kim JK Root-Specific Expression of OsNAC10 Improves Drought Tolerance and Grain Yield in Rice under Field Drought Conditions Plant Physiology 2010 153 185 197 10.1104/pp.110.154773 20335401 PMC2862432

[B34] Lu PL Chen NZ An R Su Z Qi BS Ren F Chen J Wang XC A novel drought-inducible gene, ATAF1, encodes a NAC family protein that negatively regulates the expression of stress-responsive genes in Arabidopsis Plant Molecular Biology 2007 63 289 305 17031511 10.1007/s11103-006-9089-8

[B35] Tran LSP Nakashima K Sakuma Y Simpson SD Fujita Y Maruyama K Fujita M Seki M Shinozaki K Yamaguchi-Shinozaki K Isolation and functional analysis of Arabidopsis stress-inducible NAC transcription factors that bind to a drought-responsive cis-element in the early responsive to dehydration stress 1 promoter Plant Cell 2004 16 2481 2498 10.1105/tpc.104.022699 15319476 PMC520947

[B36] Fujita M Fujita Y Maruyama K Seki M Hiratsu K Ohme-Takagi M Tran LSP Yamaguchi-Shinozaki K Shinozaki K A dehydration-induced NAC protein, RD26, is involved in a novel ABA-dependent stress-signaling pathway Plant Journal 2004 39 863 876 10.1111/j.1365-313X.2004.02171.x 15341629

[B37] Reymond P Farmer EE Jasmonate and salicylate as global signals for defense gene expression Current Opinion in Plant Biology 1998 1 404 411 10.1016/S1369-5266(98)80264-1 10066616

[B38] Xie Q Sanz-Burgos AP Guo HS Garcia JA Gutierrez C GRAB proteins, novel members of the NAC domain family, isolated by their interaction with a geminivirus protein Plant Molecular Biology 1999 39 647 656 10.1023/A:1006138221874 10350080

[B39] Yoshii M Yamazaki M Rakwal R Kishi-Kaboshi M Miyao A Hirochika H The NAC transcription factor RIM1 of rice is a new regulator of jasmonate signaling Plant Journal 2010 61 804 815 10.1111/j.1365-313X.2009.04107.x 20015061

[B40] Bu Q Jiang H Li CB Zhai Q Zhang J Wu X Sun J Xie Q Li C Role of the Arabidopsis thaliana NAC transcription factors ANAC019 and ANAC055 in regulating jasmonic acid-signaled defense responses Cell Res 2008 18 756 767 10.1038/cr.2008.53 18427573

[B41] Delessert C Kazan K Wilson IW Van Der Straeten D Manners J Dennis ES Dolferus R The transcription factor ATAF2 represses the expression of pathogenesis-related genes in Arabidopsis Plant Journal 2005 43 745 757 10.1111/j.1365-313X.2005.02488.x 16115070

[B42] Scharrenberg C Falk J Quast S Haussuhl K Humbeck K Krupinska K Isolation of senescence-related cDNAs from flag leaves of field grown barley plants Physiologia Plantarum 2003 118 278 288 10.1034/j.1399-3054.2003.00098.x

[B43] Robertson M Two transcription factors are negative regulators of gibberellin response in the HvSPY-signaling pathway in barley aleurone Plant Physiology 2004 136 2747 2761 10.1104/pp.104.041665 15347799 PMC523338

[B44] Jensen MK Rung JH Gregersen PL Gjetting T Fuglsang AT Hansen M Joehnk N Lyngkjaer MF Collinge DB The HvNAC6 transcription factor: a positive regulator of penetration resistance in barley and Arabidopsis Plant Molecular Biology 2007 65 137 150 10.1007/s11103-007-9204-5 17619150

[B45] Ogo Y Kobayashi T Itai RN Nakanishi H Kakei Y Takahashi M Toki S Mori S Nishizawa NK A novel NAC transcription factor, IDEF2, that recognizes the iron deficiency-responsive element 2 regulates the genes involved in iron homeostasis in plants Journal of Biological Chemistry 2008 283 13407 13417 10.1074/jbc.M708732200 18308732

[B46] Distelfeld A Korol A Dubcovsky J Uauy C Blake T Fahima T Colinearity between the barley grain protein content (GPC) QTL on chromosome arm 6HS and the wheat Gpc-B1 region Molecular Breeding 2008 22 25 38 10.1007/s11032-007-9153-3

[B47] Gray J Bevan M Brutnell T Buell CR Cone K Hake S Jackson D Kellogg E Lawrence C McCouch S Mockler T Moose S Paterson A Peterson T Rokshar D Souza GM Springer N Stein N Timmermans M Wang GL Grotewold E A Recommendation for Naming Transcription Factor Proteins in the Grasses Plant Physiology 2009 149 4 6 10.1104/pp.108.128504 19126689 PMC2613739

[B48] Criado MV Roberts IN Echeverria M Barneix AJ Plant growth regulators and induction of leaf senescence in nitrogen-deprived wheat plants Journal of Plant Growth Regulation 2007 26 301 307 10.1007/s00344-007-9016-5

[B49] Druka A Muehlbauer G Druka I Caldo R Baumann U Rostoks N Schreiber A Wise R Close T Kleinhofs A Graner A Schulman A Langridge P Sato K Hayes P McNicol J Marshall D Waugh R An atlas of gene expression from seed to seed through barley development Funct Integr Genomics 2006 6 202 211 10.1007/s10142-006-0025-4 16547597

[B50] Yoo SY Kim Y Kim SY Lee JS Ahn JH Control of Flowering Time and Cold Response by a NAC-Domain Protein in Arabidopsis PLoS One 2007 2 10.1371/journal.pone.0000642 PMC1920552 17653269

[B51] Jensen MK Hagedorn PH de Torres-Zabala M Grant MR Rung JH Collinge DB Lyngkjaer MF Transcriptional regulation by an NAC (NAM-ATAF1,2-CUC2) transcription factor attenuates ABA signalling for efficient basal defence towards Blumeria graminis f. sp hordei in Arabidopsis Plant Journal 2008 56 867 880 10.1111/j.1365-313X.2008.03646.x 18694460

[B52] Zhao CS Avci U Grant EH Haigler CH Beers EP XND1, a member of the NAC domain family in Arabidopsis thaliana, negatively regulates lignocellulose synthesis and programmed cell death in xylem Plant Journal 2008 53 425 436 18069942 10.1111/j.1365-313X.2007.03350.x

[B53] Guo D Liang J Li L Abscisic acid (ABA) inhibition of lateral root formation involves endogenous ABA biosynthesis in Arachis hypogaea L Plant Growth Regulation 2009 58 173 179 10.1007/s10725-009-9365-0

[B54] Guo M Rupe MA Danilevskaya ON Yang XF Hut ZH Genome-wide mRNA profiling reveals heterochronic allelic variation and a new imprinted gene in hybrid maize endosperm Plant Journal 2003 36 30 44 10.1046/j.1365-313X.2003.01852.x 12974809

[B55] Verza NC Figueira TRS Sousa SM Arruda P Transcription factor profiling identifies an aleurone-preferred NAC family member involved in maize seed development Annals of Applied Biology 2011 158 115 129 10.1111/j.1744-7348.2010.00447.x

[B56] Xue GP Bower NI McIntyre CL Riding GA Kazan K Shorter R TaNAC69 from the NAC superfamily of transcription factors is up-regulated by abiotic stresses in wheat and recognises two consensus DNA-binding sequences Functional Plant Biology 2006 33 43 57 10.1071/FP05161 32689213

[B57] Huang XQ Madan A CAP3: A DNA sequence assembly program Genome Research 1999 9 868 877 10.1101/gr.9.9.868 10508846 PMC310812

[B58] Larkin MA Blackshields G Brown NP Chenna R McGettigan PA McWilliam H Valentin F Wallace IM Wilm A Lopez R Thompson JD Gibson TJ Higgins DG Clustal W and clustal X version 2.0 Bioinformatics 2007 23 2947 2948 10.1093/bioinformatics/btm404 17846036

[B59] Paradis E Claude J Strimmer K APE: Analyses of Phylogenetics and Evolution in R language Bioinformatics 2004 20 289 290 10.1093/bioinformatics/btg412 14734327

[B60] R Development Core Team A language and environment for statistical computing R Foundation for Statistical Computing 2010 Vienna, Austria http://www.R-project.org/

[B61] Fredslund J Lange M Primique: automatic design of specific PCR primers for each sequence in a family BMC Bioinformatics 2007 8 10.1186/1471-2105-8-369 PMC2045118 17910777

[B62] Pfaffl MW A new mathematical model for relative quantification in real-time RT-PCR Nucleic Acids Research 2001 29 10.1093/nar/29.9.e45 PMC55695 11328886

[B63] Dvinge H Bertone P HTqPCR: high-throughput analysis and visualization of quantitative real-time PCR data in R Bioinformatics 2009 25 3325 3326 10.1093/bioinformatics/btp578 19808880 PMC2788924

[B64] Smyth GK Gentleman R, Carey V, Dudoit S, Irizarry R, Huber w Limma: linear models for microarray data Bioinformatics and Computational Biology Solutions using R and Bioconductor 2005 New york: Springer 397 420

[B65] Kohl M SLqPCR: Functions for analysis of real-time quantitative PCR data at SIRS-Lab GmbH. R Package, SIRS-Lab GmbH, Jena 2007

[B66] Scharrenberg C Falk J Quast S Haussuhl K Humbeck K Krupinska K Isolation of senescence-related cDNAs from flag leaves of field grown barley plants Physiologia Plantarum 2003 118 278 288 10.1034/j.1399-3054.2003.00098.x

[B67] Ogo Y Kobayashi T Itai RN Nakanishi H Kakei Y Takahashi M Toki S Mori S Nishizawa NK A novel NAC transcription factor, IDEF2, that recognizes the iron deficiency-responsive element 2 regulates the genes involved in iron homeostasis in plants Journal of Biological Chemistry 2008 283 13407 13417 10.1074/jbc.M708732200 18308732

