# Supplementary figures and images for: Characterization of barley (Hordeum vulgare L.) NAC transcription factors suggests conserved functions compared to both monocots and dicots
Source: BMC Res Notes. 2011 Aug 19;4:302. doi: 10.1186/1756-0500-4-302 (PMC3226072; doi:10.1186/1756-0500-4-302)

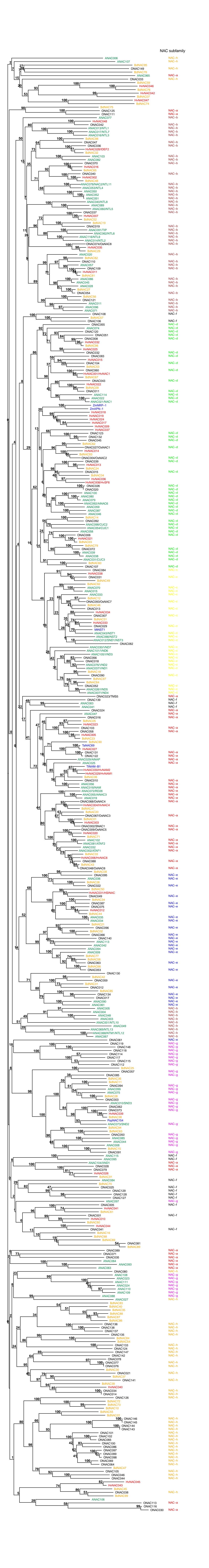

Supplement: Additional file 1 — Phylogenetic tree. Suppl_file1_Phylogenetic_tree.pdf. Phylogenetic tree of all HvNACs, BdNACs, ONACs, ANACs, and a few selected NAC genes from other species discussed in the study. [file 1756-0500-4-302-S1.PDF]
